# Enhanced Osteogenic Potential of *Noggin* Knockout C2C12 Cells on BMP-2 Releasing Silk Scaffolds

**DOI:** 10.1021/acsbiomaterials.3c00506

**Published:** 2023-10-05

**Authors:** Anıl Sera Çakmak, Sümeyra Fuerkaiti, Dilara Karagüzel, Çağatay Karaaslan, Menemşe Gümüşderelioğlu

**Affiliations:** † Department of Chemical Engineering, Hacettepe University, 06800 Ankara, Turkey; ‡ Division of Bioengineering, Graduate School of Science and Engineering, Hacettepe University, 06800 Ankara, Turkey; § Department of Biology, Molecular Biology Section, Hacettepe University, 06800 Ankara, Turkey

**Keywords:** CRISPR/Cas9, Noggin, BMP-2, silk, bone tissue engineering

## Abstract

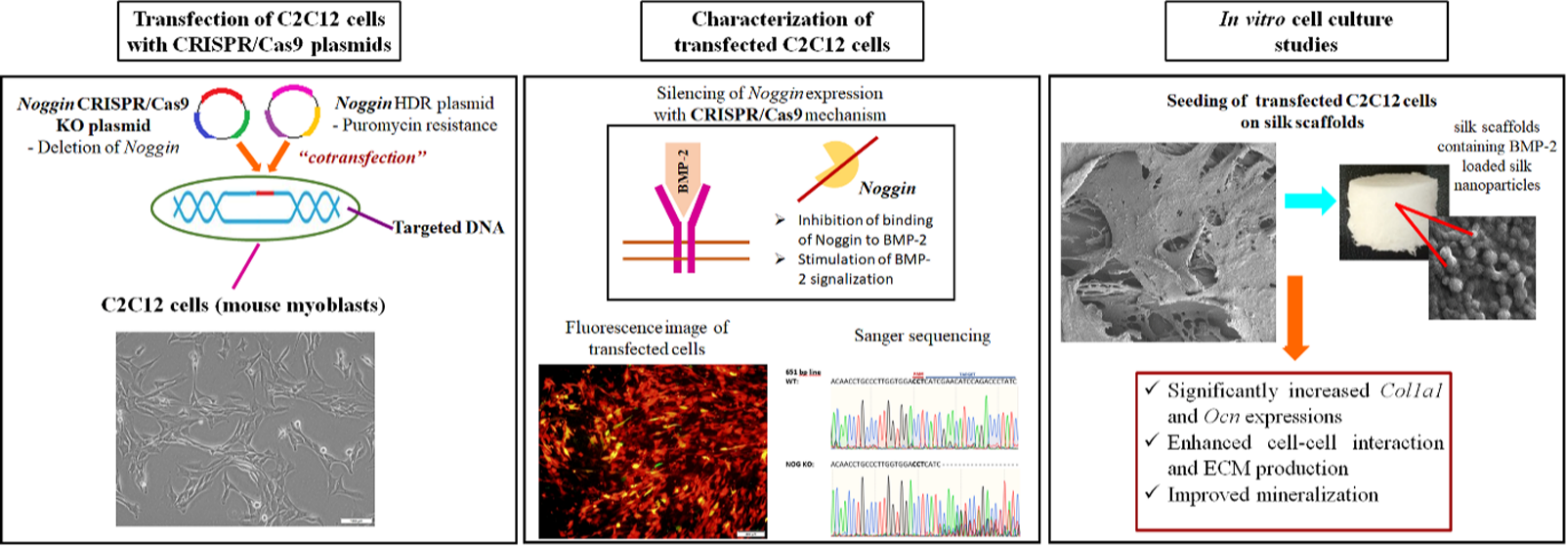

The CRISPR/Cas9 mechanism offers promising therapeutic approaches for bone regeneration by stimulating or suppressing critical signaling pathways. In this study, we aimed to increase the activity of BMP-2 signaling through knockout of *Noggin*, thereby establishing a synergistic effect on the osteogenic activity of cells in the presence of BMP-2. Since *Noggin* is an antagonist expressed in skeletal tissues and binds to subunits of bone morphogenetic proteins (BMPs) to inhibit osteogenic differentiation, here ***Noggin*** expression was knocked out using the CRISPR/Cas9 system. In accordance with this purpose, C2C12 (mouse myoblast) cells were transfected with CRISPR/Cas9 plasmids. Transfection was achieved with Lipofectamine and confirmed with intense fluorescent signals in microscopic images and deletion in target sequence in Sanger sequencing analysis. Thus, *Noggin* knockout cells were identified as a new cell source for tissue engineering studies. Then, the transfected cells were seeded on highly porous silk scaffolds bearing BMP-2-loaded silk nanoparticles (30 ng BMP-2/mg silk nanoparticle) in the size of 288 ± 62 nm. BMP-2 is released from the scaffolds in a controlled manner for up to 60 days. The knockout of *Noggin* by CRISPR/Cas9 was found to synergistically promote osteogenic differentiation in the presence of BMP-2 through increased *Coll1A1* and *Ocn* expression and mineralization. Gene editing of *Noggin* and BMP-2 increased almost 2-fold *Col1A1* expression and almost 3-fold *Ocn* expression compared to the control group. Moreover, transfected cells produced extracellular matrix (ECM) containing collagen fibers on the scaffolds and mineral-like structures were formed on the fibers. In addition, mineralization characterized by intense Alizarin red staining was detected in transfected cells cultured in the presence of BMP-2, while the other groups did not exhibit any mineralized areas. As has been demonstrated in this study, the CRISPR/Cas9 mechanism has great potential for obtaining new cell sources to be used in tissue engineering studies.

## 1 Introduction

Regulation of biological systems and organisms using molecular techniques has great potential in various fields including basic sciences, agriculture, food and pharmaceutical industries, and biotechnology. In the past 10 years, knockdown of target genes by RNA interference mechanism has been the most important development in molecular biology studies. In recent years, the clustered regularly interspaced short palindromic repeats (CRISPR)-associated protein 9 (CRISPR-Cas9) has been the most popular genome-editing system, an adaptive immune system in bacteria. In the CRISPR-Cas9 system, RNA-guided nucleases remove foreign elements from the bacteria genome and the use of the CRISPR-Cas9 system to edit animal cells and organism genomes has been described as a revolution in molecular biology. Compared to other genome-editing systems, CRISPR-Cas9 has many advantages such as easy design, high selectivity, efficiency, and multiple editing on different genes.^1^

CRISPR/Cas9 is a gene editing mechanism that allows gene knockout, activation, and repression. It has recently been used in tissue engineering for different purposes. CRISPR/Cas9-modified cells have great potential for the treatment of certain diseases such as genetic disorders,^2^ vascular disabilities,^3^ and chronic inflammation.^4^ In addition, disease models have been developed using the CRISPR/Cas9 mechanism to identify the genetic profile of diseases and to search for novel treatments.^5,6^ In cell-based studies, the CRISPR/Cas9 system supports cellular differentiation^7,8^ or enables somatic cells to gain pluripotency.^9^ Recently, CRISPR/Cas9 has been used in cartilage and bone tissue engineering studies. Truong et al. performed co-transfection with CRISPR/Cas9 plasmids for the activation of *Sox-9* and suppression of *PPAR*-γ. Transfected rat bone-marrow mesenchymal stem cells (rBMSCs) were seeded into gelatin scaffolds and chondrogenic differentiation was investigated in rat models.^10^ Ushakov et al. generated IGFBP-3 knockout human endometrial mesenchymal stem cells using CRISPR-Cas9 to improve chondrogenic differentiation.^11^ Farhang et al. achieved upregulation of *COL2A2* and *ACAN* expression by CRISPR/Cas9. They showed that glycosaminoglycan (GAG) production and collagen deposition were enhanced in the pellet culture of transfected adipose-derived mesenchymal stem cells.^12^ Hsu et al. used CRISPR/Cas9 plasmids for co-transfection of *Wnt10b* and *Foxc2*. Transfected BMSC-seeded gelatin scaffolds were placed into the calvarial defects in rats and improved bone regeneration was observed due to transfection.^13^ In another study, baculovirus system was designed for the CRISPR/Cas9 system targeting BMP-2 and *Noggin*. Gelatin scaffolds were used to deliver transfected adipose-derived stem cells to the critical size defect in rats. Overexpression of BMP-2 and inhibition of *Noggin* expression enhanced matrix mineralization.^14^

The use of exogenous growth factor is preferred in various tissue engineering studies to support tissue regeneration. On the other hand, a high dose of growth factor is required to obtain effective results, and supra-physiological high doses of these molecules may cause adverse effects on regeneration, such as abnormal tissue formation. Therefore, alternative strategies that use endogenous mechanisms such as gene editing to stimulate cells are becoming crucial. Bone morphogenetic protein (BMP) is the most studied growth factor and 20 different BMPs have been defined in humans. BMP participates in cell signaling via the SMAD pathway and takes a crucial role in ectopic cartilage and bone formation. The BMP pathway is strictly controlled in cells by intracellular and extracellular mechanisms. In the extracellular mechanism, pseudo receptors or antagonists such as “*Noggin*” bind to BMP to prevent signal formation.^15,16^ At this point, promoting osteogenic differentiation by increasing the activity of BMP-2 through *Noggin* expression constitutes an alternative approach for growth factor-based studies.^17−19^ Fuerkaiti et al. used siRNA molecules to knock down *Noggin* and investigated osteogenic differentiation of siRNA-modified cells on silk scaffolds.^20^

The CRISPR/Cas9 mechanism allows genetic modification of cells. Since these modifications occur in DNA, new properties can be transferred to the next generation of cells. Therefore, the CRISPR/Cas9 system has great potential to generate new cell sources for tissue engineering studies. Knockout of certain molecules by CRISPR/Cas9 promotes tissue regeneration, supporting cellular differentiation. We hypothesized that *Noggin* knockout cells by the CRISPR/Cas9 mechanism would show an enhanced osteogenic differentiation in three-dimensional (3D) scaffolds and could be used as a functional cell source for bone tissue engineering. In this study, expression of the BMP-2 antagonist *Noggin* in C2C12 cells was knocked out using CRISPR/Cas9 and genetically modified cells were seeded on BMP-2-loaded silk scaffolds. Within the scope of cell culture studies, proliferation, morphological changes, differentiation, and extracellular matrix (ECM) formation were monitored. In the literature, the CRISPR/Cas 9 system has been used in 2D cell cultures. In a few studies, gelatin sponge was used to deliver CRISPR/Cas9-modified cells to the damaged area in *in vivo* models. In this study, unlike other studies in the literature, the synergistic effect of decreased antagonist expression and the presence of low-dose growth factor on osteogenic differentiation was investigated in detail on nanoparticle/scaffold structures. In conclusion, the potential for the use of the developed cell/biomaterial system in bone tissue engineering was evaluated.

## 2 Materials and Methods

### 2.1 Materials

#### 2.1.1 Biomaterial Production Chemicals

*Bombyx mori* cocoons were obtained from Kozabirlik (Turkey). Sodium carbonate (Na_2_CO_3_), lithium bromide (LiBr), and sodium chloride (NaCl) were purchased from Sigma-Aldrich. Ethanol was obtained from Merck (Germany). Bone morphogenetic protein (BMP-2) and BMP-2 ELISA kit were obtained from R&D Systems and Boster Bio, respectively. **Transfection chemicals:** control CRISPR plasmid, CRISPR KO (knockout) & homology-directed repair (HDR) plasmids, puromycin, and transfection medium were purchased from Santa Cruz. Lipofectamine3000 kit was obtained from Thermo Fisher Scientific. **Cell culture chemicals:** Dulbecco’s modified Eagle’s medium-high glucose (DMEM-high glucose), fetal bovine serum (FBS), and Dulbecco’s phosphate-buffered saline (DPBS) were obtained from Capricorn (Germany). l-Glutamine, penicillin/streptomycin (P/S) solution, and trypsin-EDTA were purchased from Sigma-Aldrich. Thiazolyl blue tetrazolium bromide (MTT) salt was purchased from AppliChem. Isopropanol was obtained from Merck (Germany). Trizol and RNeasy kit were obtained from Qiagen. High-capacity cDNA reverse transcription kit, 5× Hot FIREPol EvaGreen qPCR mix plus, and calcium colorimetric assay kit were purchased from Applied Biosystems, Solis Biodyne (Estonia), and BioVision, respectively. Glutaraldehyde and hexamethyldisilazane (HMDS) were obtained from Sigma-Aldrich. Also, paraformaldehyde and xylene were purchased from Sigma-Aldrich. Alizarin red was obtained from Thermo Fisher Scientific.

### 2.2 Methods

#### 2.2.1 Fabrication and Characterization of Silk Scaffolds

*B. mori* cocoons were used to produce silk fibroin solution using the standard protocol. Briefly, small cocoon pieces were boiled in Na_2_CO_3_ solution (0.02 M) for 30 min, then washed 3 times with ultrapure water, and dried at room temperature to obtain fibroin extract. The extract was dissolved in 9.3 M LiBr solution at 60 °C for 4 h, then the fibroin solution was kept in ultrapure water for 2 days in the dialysis membrane, and centrifuged twice at 4500*g* for 20 min. To fabricate silk scaffolds, silk solution (5 mL, 6% w/v) was poured into a Teflon mold with NaCl particles (500–700 μm, 10 g). After 48 h, silk scaffolds were soaked in ultrapure water for 48 h and water was changed several times in a day to remove salt particles.^21,22^ The scaffolds (diameter: 6 mm, thickness: 2 mm) were fabricated via freeze-drying (Christ, Germany) at −80 °C. Autoclave sterilization was applied to the scaffolds before *in vitro* studies. Morphologic examination of the scaffolds was performed using a scanning electron microscope (SEM) (GAIA3 TESCAN, Czechia) and pore sizes of the scaffolds were measured via ImageJ software (NIH). To calculate the porosity and water-uptake capacity of silk scaffolds, the gravimetric method described in our previous work was used.^20^

#### 2.2.2 Production and Characterization of Silk Nanoparticles

Silk nanoparticles were prepared by “*self-assembling*” method.^23^ First, ethanol was slowly added to the silk fibroin solution (3% w/v) at 25 °C under magnetic stirring, with a final volume ratio of 6:20 (*V*_ethanol_/*V*_silk_). The solution was centrifuged at 13,000 rpm for 30 min after storing at −20 °C overnight. Once the supernatant was removed, nanoparticles were washed with ultrapure water. The size and size distribution of particles were determined by dynamic light scattering (Malvern, England) and observed by SEM.

#### 2.2.3 Loading and In Vitro Releasing of BMP-2

Growth factor-loaded nanoparticles were prepared by using the method described by Bessa et al.^24^ In brief, BMP-2 was added to the silk fibroin solution at a concentration of 6.4 μg BMP-2/mL and the final solution was stirred at 600 rpm at room temperature. Then, the self-assembling protocol given above was followed. For the determination of encapsulation efficiency, the supernatant was collected after washing steps and BMP-2 in the collected solution was analyzed by using the ELISA kit. After the washing step, BMP-2-loaded nanoparticles were embedded into the silk scaffolds (0.1 mg particles/scaffold) and the scaffolds were left in an incubator at 37 °C to dry. For release studies, nanoparticle-embedded scaffolds were placed in 1 mL of PBS (pH:7.4) and incubated at 37 °C. At the predetermined time points 1 mL of solution was removed, centrifuged, and stored at −20 °C until analysis. The volume of extracted solution was replaced with fresh water. The cumulative release ratio was calculated using the ELISA kit.

#### 2.2.4 Transfection Studies

Transfection studies were performed with mouse myoblast C2C12 cells at passage 4 to 8 (DSMZ ACC 565; Germany) cultured in DMEM-HG medium containing 10% FBS and 1% P/S (growth medium) in a CO_2_ incubator (Heraeus Instruments, Germany).

##### 2.2.4.1 Determination of Transfection Parameters on C2C12 Cells

The amount of Lipofectamine3000 (3, 4, 6 μL/mL) and the transfection period (24 and 48 h) were changed to determine the transfection efficiency. C2C12 cells were seeded into 48-well plates (2.5 × 10^4^ cells/well) in the growth medium without antibiotic (0.25 mL/well). Transfection was carried out using the cells with 60–70% confluency (approx. 24 h later). According to the manufacturer’s procedure, 0.1 μg/μL control CRISPR plasmid was used as a stock solution. Briefly, 2.5 μL of control CRISPR plasmid and different concentrations (2, 2.68, 4 μL/mL) of **P3000** (transfection enhancer reagent in Lipofectamine3000 kit) were completed to 12.5 μL with transfection medium to obtain **Solution A**. To obtain **Solution B**, different concentrations (3, 4, 6 μL/mL) of **Lipofectamine3000** solutions were made up to 12.5 μL with transfection medium. Solution A was added drop by drop to solution B in a ratio of 1:1 to form the transfection solution (**Solution A + B**) and it was kept at room temperature for 20 min, and then 25 μL of transfection solution was added into the growth medium for each well. The fluorescence microscope was used to observe the transfection at 24 and 48 h after transfection. Transfection conditions of C2C12 cells are given in Table 1.

**Table 1:** Determination of Transfection Conditions of C2C12 Cells with Control CRISPR Plasmid

passage	number of cells (per well)	control CRISPR plasmid concentration (per well)	lipofectamine3000 concentration (per well)	transfection solution treatment time (h)	well plate	culture medium (per well)
C2C12 (P.5)	2.5 × 10^4^	1 μg/mL	3 μL/mL	24 h or 48 h	48 well	250 μL
			4 μL/mL			
			6 μL/mL			

##### 2.2.4.2 Determination of Puromycin Selection Parameters on C2C12 Cells

C2C12 cells were seeded into 48-well plates with a density of 2.5 × 10^4^ cells/well and cultured in the growth medium without antibiotic (0.25 mL/well). Transfection was performed using the cells with 60–70% confluency (approx. 24 h later). Briefly, 2.5 μL of CRISPR KO plasmid, 2.5 μL of HDR plasmid, and 0.5 μL of P3000 were mixed into the 7 μL transfection medium to obtain **CRISPR plasmid solution**. **Lipofectamine3000 solution** was prepared by adding 1.5 μL of Lipofectamine3000 to 11 μL of transfection medium. Then, CRISPR plasmid solution was added drop by drop to Lipofectamine3000 solution (**final transfection solution**), and it was left at room temperature for 20 min. Finally, 25 μL of the final transfection solution was added to each well. The medium was removed after 48 h of transfection, and 250 μL of fresh growth medium with different amounts of puromycin (2 and 4 μg/mL) was added to the wells. We investigated the puromycin selection efficiency by MTT assay on the fourth day and microscopic images. Selection conditions of transfected C2C12 (t-C2C12) cells with puromycin are given in Table 2.

**Table 2:** Transfection and Puromycin Selection Conditions of C2C12 Cells

passage	number of cells (per well)	HDR plasmid concentration (per well)	KO plasmid concentration (per well)	lipofectamine3000 concentration (per well)	transfection solution treatment time (h)	puromycin concentration (per well)	puromycin treatment time (d)	well plate	culture medium (per well)
C2C12 (P.6)	25 × 10^4^	1 μg/mL	1 μg/mL	6 μL/mL	48 h	2 μg/mL	4 day	48 well	250 μL
						4 μg/mL			

##### 2.2.4.3 Generation of Transfected C2C12 Cells (t-C2C12)

Transfected C2C12 cells were obtained in 6-well plates (2.5 × 10^5^ cells/per well) using the transfection protocol described above. Since the transfection conditions were optimized in 48-well plates, the amounts of transfection reagents were changed according to the surface area of the 6-well plate. The medium was discharged after 48 h of transfection and 2 mL of fresh growth medium containing 4 μg/mL puromycin was added to the wells. Cells were then incubated at 37 °C for 4 days. The puromycin medium was refreshed every two days, and after 4 days it was completely removed, and fresh growth medium was added to the cells. After the cells reached confluency, trypsinization was performed to obtain transfected cell stocks.

Sanger sequencing was performed to confirm *Noggin* knocked out cells after transfection. Extraction of genomic DNA was completed with QIAmp DNA Mini Kit (Qiagen) following the manufacturer’s protocol. The DNA quality was investigated using a Qubit 3 fluorometer (Thermo Fisher Scientific). The wild-type (WT) and CRISPR-edited *Noggin* genes were amplified using Nog-F:5′-GTCGCGCTGGAGTAATCTCG-3′ and Nog-R:5′-GCGCTGTTTCTTGCCTTGG-3′ primers. PCR amplification was performed with OneTaq Hot Start DNA Polymerase (New England Biolabs) in Applied Biosystems SimpliAmp Thermal Cycler (Thermo Fisher Scientific). Amplicons were purified by Exonuclease I and Shrimp Alkaline Phosphatase enzymes (Thermo Fisher Scientific). A sequencing reaction was performed using a Big Dye Terminator v3.1 Cycle Sequencing Kit (Thermo Fisher Scientific) with an ABI 3500 Genetic Analyzer (Thermo Fisher Scientific). The results were analyzed with SnapGene (GSL Biotech LLC) and Blast (NCBI).

#### 2.2.5 In Vitro Studies with Transfected Cells

*In vitro* studies were performed in 24-well plates. Non-transfected (control) and transfected C2C12 (t-C2C12) cells were seeded onto the sterile silk scaffolds (8 × 10^4^ cells/scaffold). Cell culture studies were carried out in 1 mL/well growth medium (DMEM-HG containing 10% v/v FBS and 1% P/S), and it was continued for 21 days in a CO_2_ incubator at 37 °C with the medium refreshed every 3 days. Experimental groups were as follows:

(1) C2C12 (control): silk scaffolds seeded with non-transfected cells.
(2) C2C12/BMP-2: silk scaffolds carrying BMP-2-loaded silk nanoparticles seeded with non-transfected cells.
(3) t-C2C12: silk scaffolds seeded with transfected cells.
(4) t-C2C12/BMP-2: silk scaffolds carrying BMP-2-loaded silk nanoparticles seeded with transfected cells.

##### 2.2.5.1 Cell Activity (MTT Assay)

The cell activity of transfected and non-transfected C2C12 cells on silk scaffolds was evaluated with MTT assay. Culture medium was removed at days 2, 7, 14, and 21, and scaffolds were transferred to another plate after washing with DPBS (pH: 7.4). Then, 300 μL of freshly prepared DMEM-HG containing 10% MTT solution (2.5 mg/mL in PBS) was added to each scaffold. After incubation for 3 h at 37 °C, the medium was replaced with 200 μL of isopropanol solution. Then, absorbance of the supernatant was measured at 570 nm (reference:690 nm) using a microplate reader (Asys UVM 340, Austria).

##### 2.2.5.2 Gene Expression Analysis (qPCR)

The cell-seeded scaffolds were transferred to microtubes after washing with DPBS. They were then cut into pieces with a microscissor. The mRNA isolation was performed in Trizol using the RNeasy mini column kit (Qiagen) protocol. Quality of mRNA was evaluated by using a Nanodrop 2000 (Thermo Fisher Scientific). Then, cDNA was transcribed from the mRNA using high-capacity cDNA reverse transcription kit with a thermal cycler (Thermo Fisher Scientific). Expression levels of mouse Collagen type I (*Coll I*) and Osteocalcin (*Ocn*) were detected via qPCR (LightCycler Nano, Roche, Switzerland). PCR reaction mixture was prepared according to the protocol of 5× HOT FIREPol EvaGreen qPCR mix. Quantitative polymerase chain reactions were performed for 45 cycles at 95 °C for 15 s, at 52 °C for 20 s, and at 72 °C for 20 s. Evaluation of gene expression in the cells was carried out using the 2^–ΔΔCT^ method and β-*Actin* was chosen as a control primer to normalize expression levels of target genes. Information about primers is given in Table 3.

**Table 3:** Primer Sequences and Melting Temperatures (*T*_m_) of Genes Used in q-PCR Analysis

genes	forward primer	reverse primer	*T*_m_ (°C)
β-*Actin*	F-GCCCTGAGGCTCTTTTCCAG	R-TGCCACAGGATTCCATACCC	57
*Col1a1*	F-CAAGATGTGCCACTCTGACT	R-TCTGACCTGTCTCCATGTTG	52
*Ocn*	F-CTTTCTGCTCACTCTGCTG	R-TATTGCCCTCCTGCTTGG	52

##### 2.2.5.3 Cell Morphology (SEM)

The morphology of C2C12 cells on silk scaffolds was observed by SEM (GAIA3 TESCAN, Czechia) at days 7 and 21. First, the culture medium was discharged from the scaffolds, and they were washed three times with DPBS. Cells were then fixed with glutaraldehyde solution (2.5%, v/v) for 30 min at 4 °C. Dehydration of the samples was carried out in graded ethanol series for 2 min each. Finally, the samples were treated with HMDS for 5 min and dried overnight. Before analysis, the scaffolds were coated with gold and palladium.

##### 2.2.5.4 Mineralization (Alizarin Red Staining)

At day 21, the samples were fixed with 4% paraformaldehyde at 4 °C overnight. Graded ethanol series (70, 95, 100%, 30 min) were used for dehydration of samples. After treatment with xylene for 30 min, samples were embedded in paraffin. Then, 8 μm sections were cut via a microtome (Leica Biosystems, Germany) and samples were taken to the lamella. Alizarin red staining (5 min) was applied to the de-paraffinized samples and an optical microscope was used to visualize the samples (Olympus, Japan).

## 3 Results and Discussion

In recent years, molecular biology techniques have been preferred to modify cells in tissue engineering studies and small RNA molecules are generally used for gene editing. In our previous study, *Noggin* was knocked down in MC3T3-E1 cells using siRNA molecules and it was found that the osteogenic differentiation of these cells on silk scaffolds was improved as a result of *Noggin* suppression.^20^ However, siRNA-based applications target protein synthesis, and its effect is temporary, while the CRISPR/Cas9 system directly edits the genome, allowing long-term modification. Therefore, we decided to conduct the present study with the CRISPR/Cas9 system.

### 3.1 Fabrication and Characterization of Silk Scaffolds

**Figure 1:**
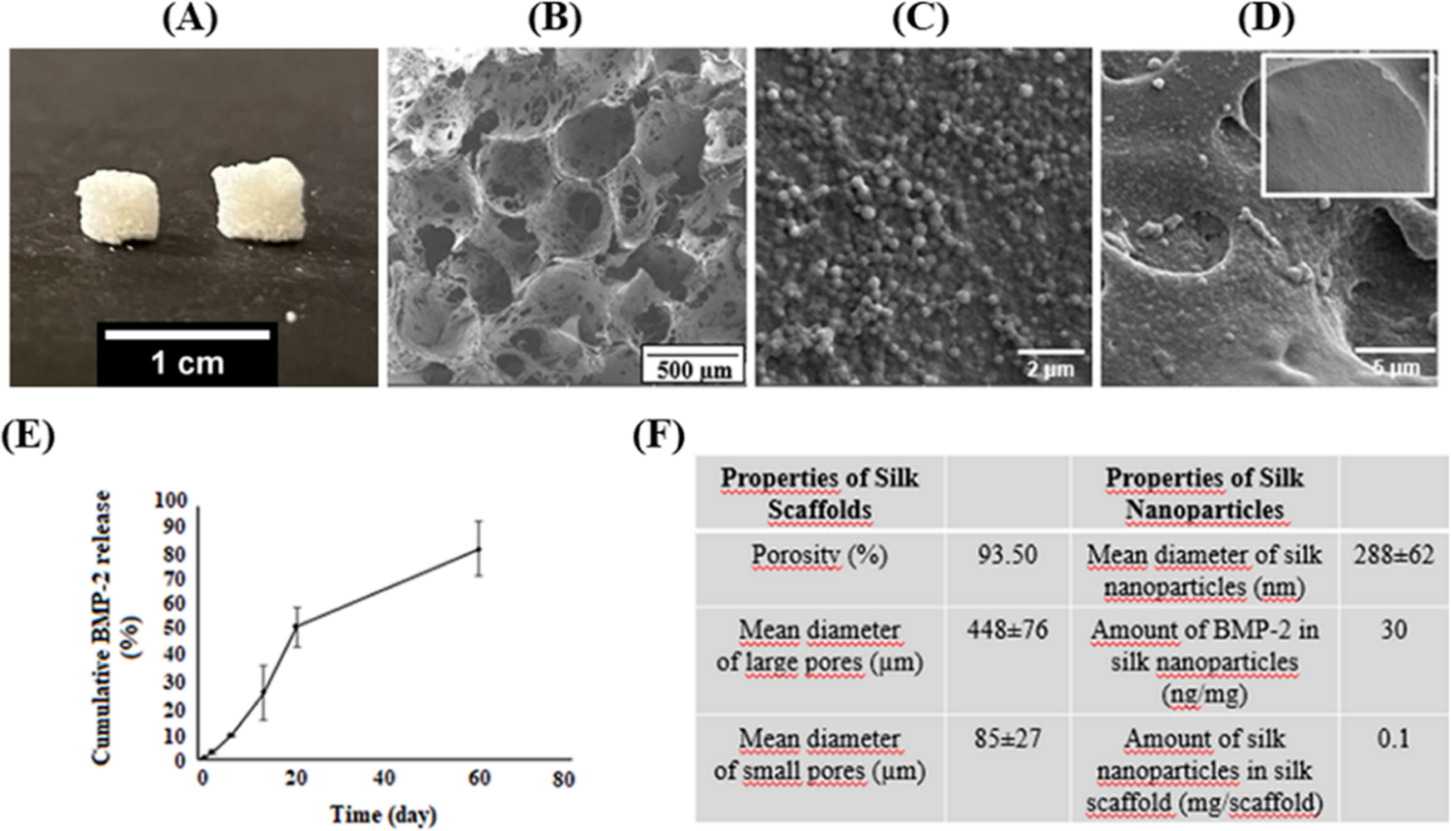
(A) Macroscopic and (B) microscopic images of silk scaffolds, (C) SEM images of silk nanoparticles, (D) silk nanoparticle-embedded scaffold (empty scaffold on the upper side), (E) cumulative BMP-2 release from silk nanoparticle-embedded scaffolds, and (F) properties of silk scaffolds and nanoparticles.

Silk-based scaffolds have enormous advantages for bone regeneration, such as good biocompatibility, slow degradation, excellent mechanical strength, and inducing biomineralization. Moreover, the silk peptide allows for a different scaffold design.^25^ In this study, silk scaffolds were obtained in sponge form by using solvent-casting/particulate-leaching method (Figure 1a) and salt (sodium chloride) was used as a porogen.^26−28^ SEM images in Figure 1b show that salt leaching enabled the production of scaffolds with high porosity (93.5 ± 0.02%) in different-sized pores homogeneously dispersed and efficient interconnectivity. While large pores (448 ± 76 μm) are favorable for cell migration, small pores (85 ± 27) are suitable for nutrient-metabolite transport. In swelling studies, silk scaffolds reached the equilibrium swelling in PBS at 37 °C in the first 5 min and preserved their form without dissolving in water.

### 3.2 Production and Characterization of BMP-2-Loaded Silk Nanoparticles

Silk nanoparticles have been prepared by using different methods, such as desolvation,^29^ soft template formation,^30^ and solution-enhanced dispersion using supercritical carbon dioxide (SEDS).^31^ Unlike these methods, the self-assembling method is performed in mild conditions without any initiators, cross-linking agents, or organic solvents. Therefore, in this method, activity of growth factors is preserved.^32^ Silk concentration, type and concentration of alcohol, and freezing temperature are important parameters that affect particle shape, size, and size distribution.^23^

In this study, BMP-2-loaded nanoparticles were successfully produced by using the parameters reported by Cao et al., 3% (w/v) silk fibroin concentration, −20 °C freezing temperature, and 6:20 ethanol ratio.^23^Figure 1c shows the spherical morphology of silk nanoparticles without apparent aggregation. Polydispersive index (PDI) was 0.364 ± 0.013 according to Zeta sizer analysis. PDI describes the degree of non-uniformity and size distribution of nanoparticles in a suspension. A PDI value close to 0 indicates monodisperse particles, while a value close to 1 indicates a very broad particle size distribution. The average size of uniform nanoparticles was calculated as 288 ± 62 nm by using ImageJ.

Bone morphogenetic protein plays a crucial role in osteogenesis, and sustained release of BMP-2 has shown significant therapeutic potential for bone regeneration.^33^ In this study, BMP-2 was added to the silk solution at a concentration of 30 ng BMP-2/mg particle during the production of silk nanoparticles, and they were incorporated into the silk scaffold at a concentration of 0.1 mg particles/per scaffold. As seen in Figure 1d, silk nanoparticles strongly adhered to the interior of the scaffold and their homogenous distribution throughout the entire scaffold increased the roughness of the surface compared to the control group (on the upper side of Figure 1d).

The release study was conducted in PBS at 37 °C and BMP-2 release kinetics is shown in Figure 1e. Silk is a favorable material for controlled release as a result of its high binding capacity, sustained release profile, and mechanical stability that avoids loss of bioactivity of biomolecules.^34,35^ During the formation of silk nanoparticles, the structure of the silk changes to α-helix and random coil to highly crystalline β-sheets. This conformational transition provides good resistance to dissolution and prevents growth factors from thermal and enzymatic degradation. Additionally, the growth-factor release mechanism of silk nanoparticles is the diffusion of biomolecules through the degraded polymeric matrix.^25^ The release of BMP-2 from silk nanoparticles initially showed a linear profile without a burst effect. It was determined that almost 50% of the loaded BMP-2 was released up to 20 days. (Figure 1e). Silk protein has a wide variety of amino acids with functional groups that allow binding with different biomolecules.^25^ Several studies have reported that electrostatic interactions occur between silk protein and growth factors.^36,37^ On the other hand, using scaffolds to carry nanoparticles ensures targeted drug delivery and decreases the release rate of biomolecules by increasing the diffusion distance.^25,37^ As shown in Figure 1e, the initial BMP-2 release was followed by a more sustained and slower release until day 60. All properties of silk scaffolds and nanoparticles are given in the table in Figure 1f.

### 3.3 Transfection Studies

**Figure 2:**
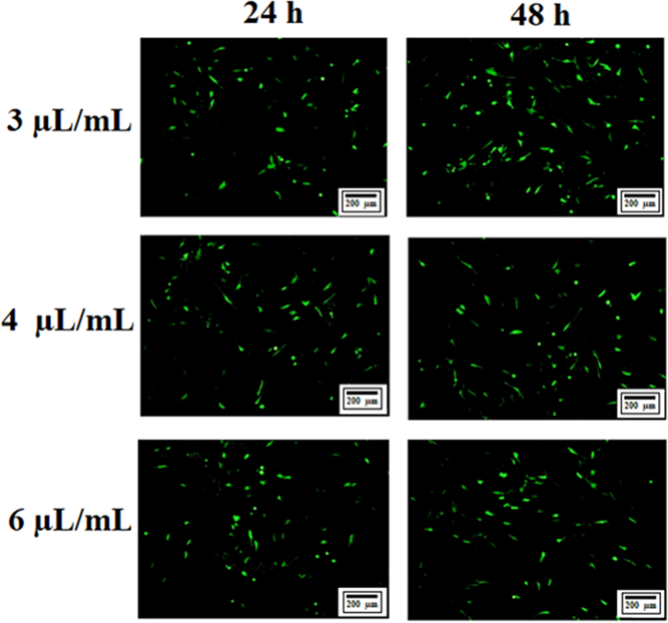
Optical microscopic images showing the transfection results of C2C12 cells with different Lipofectamine3000 concentrations and two different transfection times.

**Figure 3:**
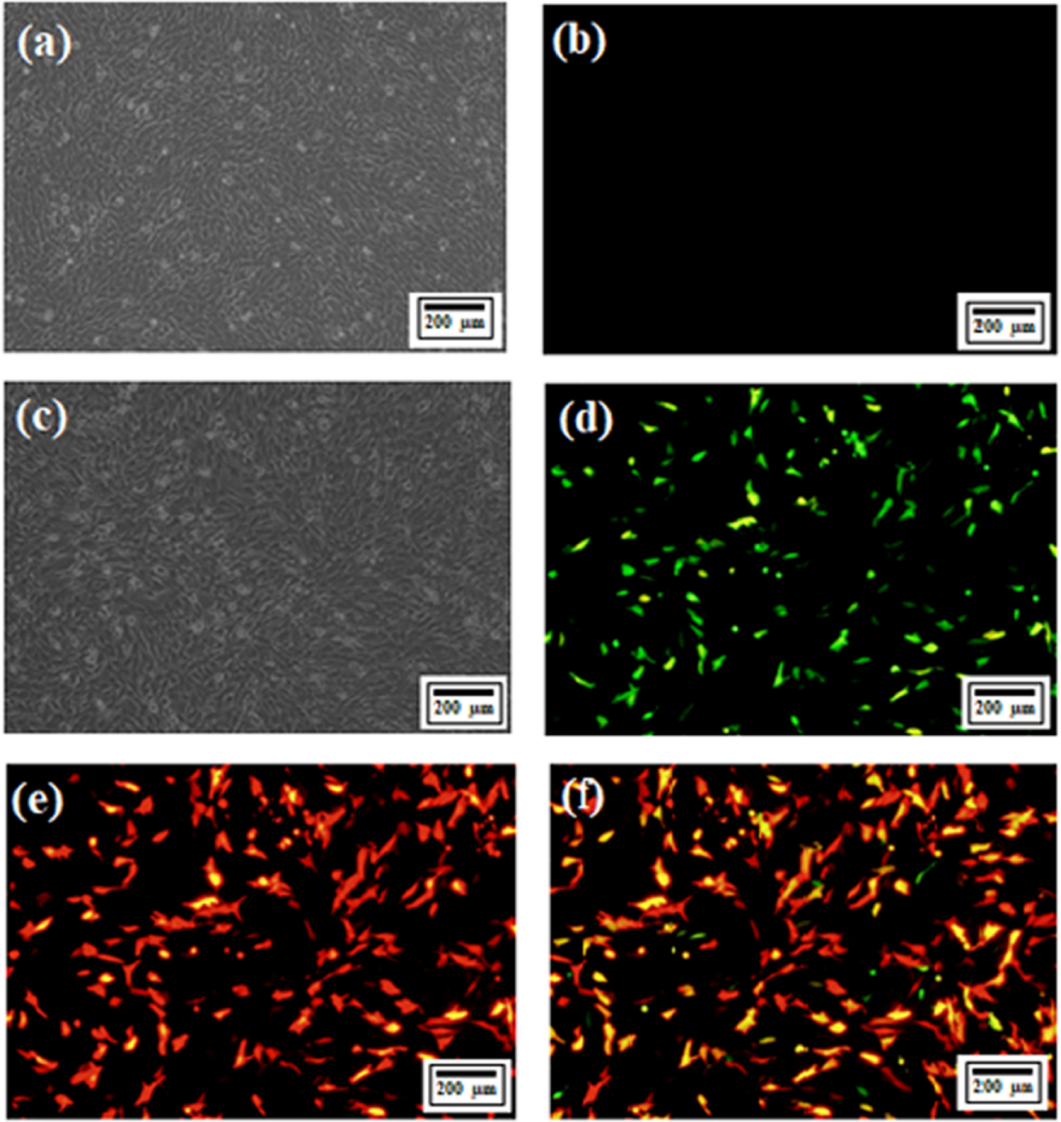
Transfection results with *Noggin* CRISPR/Cas9 KO and HDR plasmids. (a) Optical microscopic image of C2C12 cells, (b) fluorescence microscopic image of C2C12 cells, (c) optical microscopic image of t-C2C12 cells, (d) GFP fluorescence in culture transfected with the CRISPR/Cas9 KO plasmid, (e) RFP fluorescence in culture transfected with the HDR plasmid, and (f) merged image of GFP and RFP fluorescence.

Transfection studies were carried out with C2C12 cells, the muscle precursor cell line. It is known that differentiation pathway of C2C12 alters in osteogenic lineage in the presence of BMP-2. BMP-2 not only induces osteogenesis but also inhibits the differentiation of C2C12 into mature muscle cells.^38,39^ C2C12 cells are preferred as a cell source in transfection studies due to their high transfection efficiency (∼70–80%).^40,41^ In recent studies, C2C12 cells have been transfected with CRISPR/Cas9 plasmids to elucidate myogenic differentiation pathways^42,43^ or to investigate protective mechanisms in cells under oxidative stress.^44^ Within the scope of transfection studies, control plasmid was used primarily to determine the experimental condition. According to the protocol, concentration of the control plasmid was 1 μg/mL for each well. Lipofectamine3000 concentration was changed to 3, 4, and 6 μL/mL and two different transfection times, 24 and 48 h, were applied. The control plasmid contained green fluorescence protein (GFP), so the transfection efficiency was visualized under a fluorescence microscope. As seen in Figure 2, the transfection efficiency was enhanced with the prolongation of the application time. Besides, the number of GFP-fluorescent cells was increased in the presence of 3 μL/mL Lipofectamine3000 compared to other groups. Therefore, 3 μL/mL Lipofectamine3000 and 48 h application time were chosen as transfection parameters for *Noggin* CRISPR/Cas9 KO and *Noggin* HDR plasmids. Knockout and HDR plasmids contained GFP and red fluorescence protein (RFP), respectively, to visually verify transfection. After transfection, transfected cells exhibited intense green and red signals, indicating successful entry of both plasmids into the cells. On the other hand, there was no signal in the control group (Figure 3).

**Figure 4:**
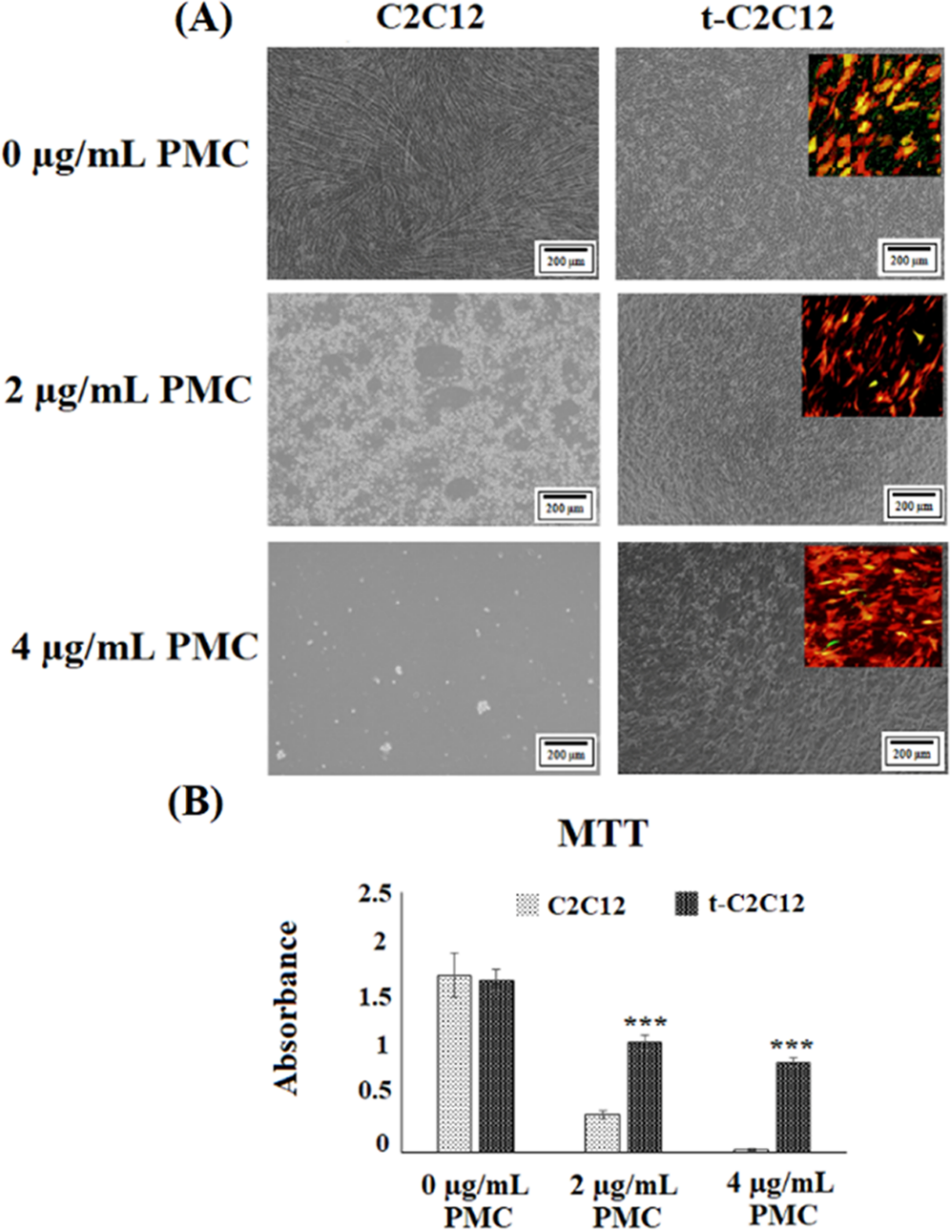
Puromycin selection studies: (A) optical microscopic images of C212 and t-C212 cells in the presence of 0, 2, and 4 μg/mL puromycin. Fluorescence microscopic images of t-C2C12 cells are in the upper right side of the optical microscopic images. (B) MTT analysis was done to determine cell viability after puromycin selection.

Forty–eight hours after transfection, 2 and 4 μg/mL puromycin (PMC) was added to the cell culture medium to select transfected cells. The *Noggin* KO plasmid has a specific RNA sequence, which is a guideline for Cas9 to disrupt target gene expression by causing double-strand break in DNA. On the other hand, *Noggin* HDR plasmids involved in DNA repair have puromycin-resistant gene, allowing selection of Cas9-induced DNA damaged cells. Optical microscopic images of cells and MTT analysis are shown in Figure 4. Cells without any transfection and PMC addition (control group) adhered strongly to the cell culture surface, spread well, and proliferated densely on the surface. Transfected cells proliferated on the surface and reached confluency, similar to the control group. Also, cells had fluorescence signals as a result of transfection and there was no cytotoxic effect of transfection on cells. In the presence of 2 μg/mL PMC, the flattened morphology of control cells turned into spherical form and cells began to detach from the surface. Moreover, cells in the control group completely detached from the surface after the addition of 4 μg/mL PMC. Cell viability for transfected cells was maintained in both 2 and 4 μg/mL PMC groups. In addition, more transfected cells with intense fluorescence signals were identified in the presence of 4 μg/mL PMC compared to other groups.

MTT analysis showed similar results with optical images. In the absence of PMC, there was no difference in cell viability between control (non-transfected) and transfected cells. Although the addition of puromycin slightly decreased the cell viability in transfected cells, cellular activity of these cells was significantly higher than that in the control group. Especially for the 4 μg/mL PMC groups, cells showed pronounced cellular activity after transfection, whereas control cells had almost no cellular activity. Based on these results, PMC concentration was determined as 4 μg/mL to select the transfected cells more effectively and purely. Puromycin selection results demonstrated that knockout of the *Noggin* gene was achieved in C2C12 cells by co-transfection of these plasmids under the experimental conditions mentioned here.

**Figure 5:**
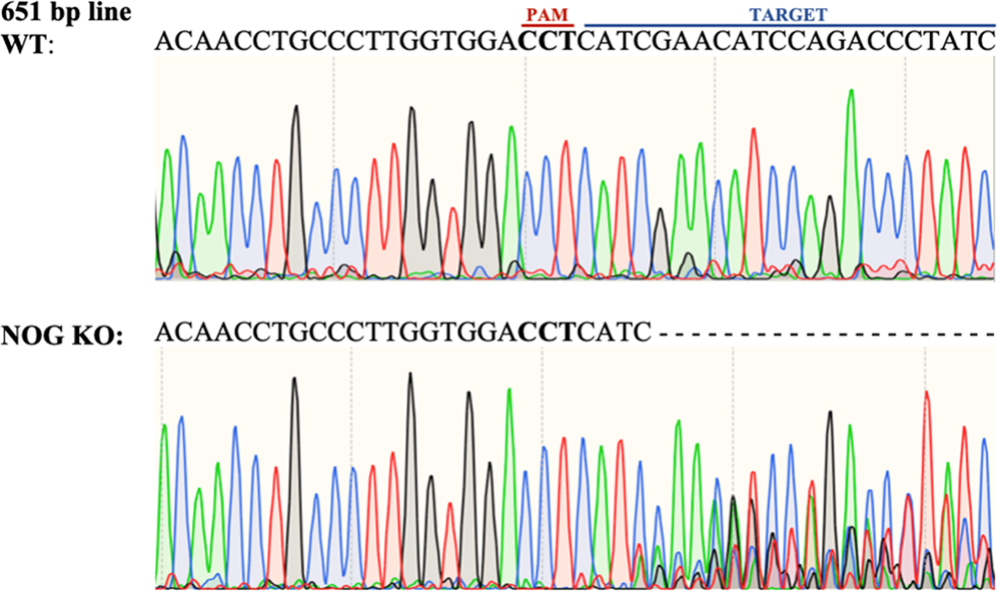
Sanger sequencing chromatograms of the wild-type (WT) and NOG KO. The PAM site (bold) and predicted cut site (TARGET) are shown.

In addition, Sanger sequencing was used to confirm the knockout of *Noggin* gene in the CRISPR/Cas9-transfected cells. The *Noggin* gene site covering deleted sequences was amplified, and amplification products were controlled using agarose gel electrophoresis (data not shown). Compared to the WT sequence, gene sequencing results of transfected cells showed that the *Noggin* gene was edited by the *Noggin*-CRISPR/Cas9 KO system after the PAM sequence (Figure 5).

The sequence of *Noggin* gene (651 bp) was compared between WT and NOG KO samples. After PAM sequence site, which was located at 367 bp, there was no deletion observed in WT samples. On the other hand, frameshift was detected after the PAM sequence in NOG KO samples.

Three different sgRNA sequences were used during Noggin-CRISPR/Cas9 knockout experiments. These sequences resulted in the cleavage of 3 different bases and a frameshift mutation in the “TARGET” site. This situation was observed as mixed trace signals in the NOG KO chromatogram. Similar chromatograms were seen in studies that used the Sanger sequencing method to confirm the success of CRISPR/Cas9.^45,46^ According to the findings in these studies, Sanger sequencing results confirmed that *Noggin* gene was successfully knocked out by the CRISPR/Cas9 method in NOG KO samples.

### 3.4 In Vitro Studies with Transfected Cells

Cell culture studies were conducted with control and transfected cells, and proliferation, morphology, ECM production, and osteogenic differentiation were monitored for 21 days.

#### 3.4.1 MTT Analysis

**Figure 6:**
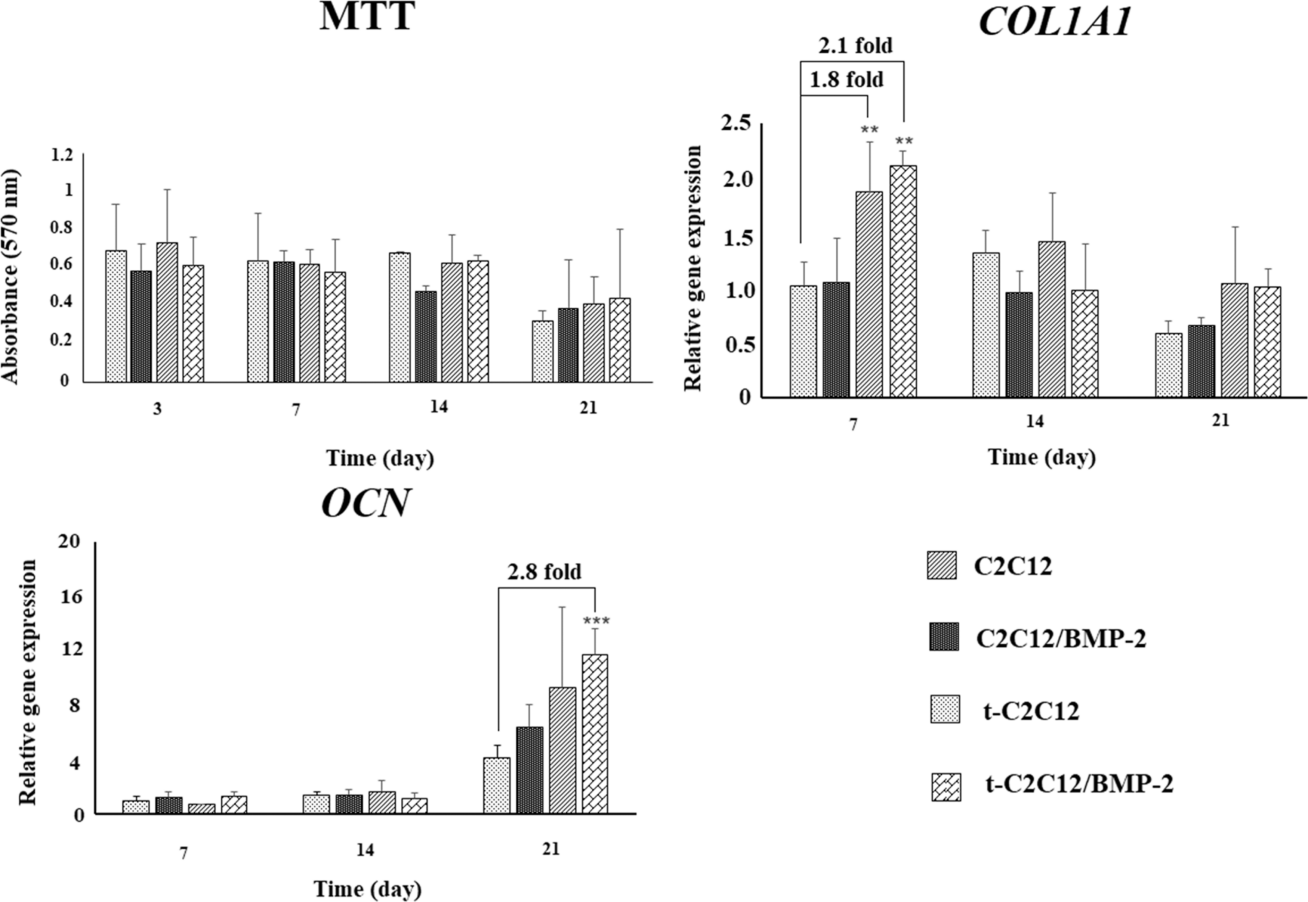
Cell viability (MTT results) and relative gene expressions of C2C12 and t-C2C2 cells on the silk scaffolds and BMP-2-loaded silk scaffolds (*p < 0.05, **p < 0.01, ***p < 0.001, *n* = 3).

Cell proliferation in the scaffolds was evaluated using the MTT assay (Figure 6). As seen in the MTT graph, cell proliferation remained stable throughout the cell culture period. On day 21, the mitochondrial activity slightly decreased in the control group while it did not change in other groups. Also, there was no significant difference among the groups (*p ≥ 0.05). Therefore, no transfection-related inhibition on cell proliferation was determined.

#### 3.4.2 qPCR Analysis

The osteogenic differentiation of cells seeded on silk scaffolds was investigated molecularly using qPCR. As seen in Figure 6, *Coll1a1* expression in transfected cells was higher (approximately 2-fold) than that in the control group at day 7 (**p < 0.01). Transfection also stimulated *Coll1a1* expression compared to the C2C12/BMP-2 group. Besides, transfection in the presence of BMP-2 increased *Coll1a1* expression (*p < 0.01) more than transfection alone (*p < 0.05). Collagen expression decreased in transfected cells on day 14, while it showed a stable profile in non-transfected cells. It was also reduced in control groups from day 14 to day 21. Collagen is an important structural protein located in the ECM. In the literature, *Coll1a1* expression was found to be higher in the proliferation phase and at the beginning of ECM production, and it was downregulated in the mineralization phase. So, it has been defined as an early-stage osteogenic marker during osteogenic differentiation.^47,48^

Osteocalcin expression is also shown in Figure 6. The expression level of *Ocn* reached the maximum level in all groups on day 21. At day 21, the *Ocn* expression level was highest in the t-C2C12/BMP-2 group. Transfection and BMP-2 synergistically increased *Ocn* expression almost 3-fold compared to the control group (***p < 0.001). At the same time, *Ocn* expression in this group was higher than that in control cells cultured in the presence of BMP-2 (**p < 0.01). Osteocalcin is secreted by terminally differentiated osteoblasts and is responsible for mineralization and bone matrix formation.^49^ Mineralized nodule formation is characterized by sequentially increased expression of alkaline phosphatase (ALP), bone sialoprotein (BSP), and *OCN*.^50^ Thus, *Ocn*, the most abundant non-collagenous protein in bone matrix, is a late-stage marker for osteogenic differentiation.^51^

#### 3.4.3 SEM Analysis

**Figure 7:**
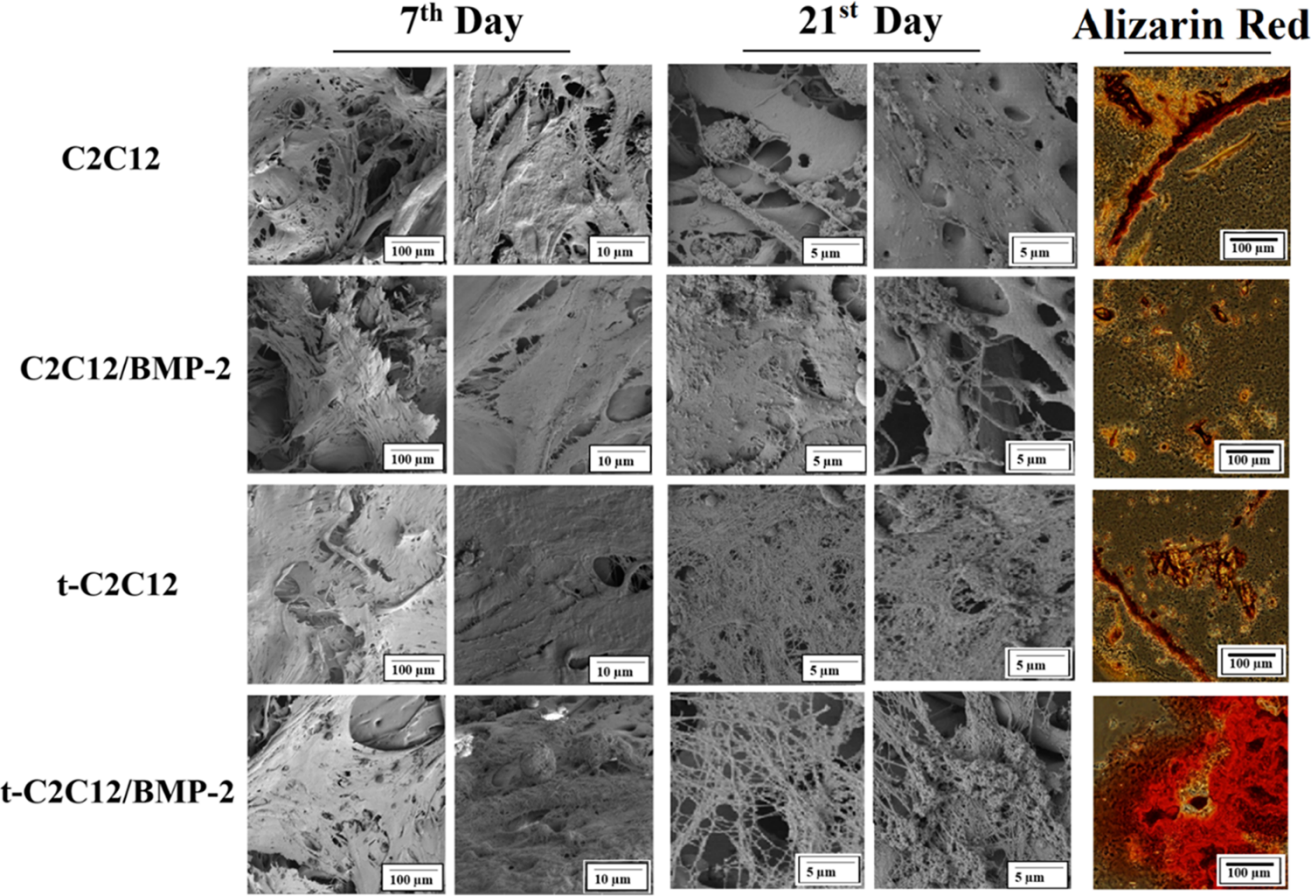
SEM images of C2C12 and t-C2C2 cells on to the silk and BMP-2-loaded silk scaffolds. Alizarin red staining was done to determine mineralization. Alizarin red staining indicates the deposition of calcium with dark red precipitates.

Cellular distribution in the scaffolds, cell–cell and cell–scaffold interactions, and ECM formation were investigated using SEM (Figure 7). On day 7, cells successfully attached and spread on to the scaffolds in all groups. Transfected cells exhibited more intense ECM formation with collagen-like fiber structures. On day 21, ECM production characterized with collagen fibers increased in transfected groups. Moreover, transfected cells in the presence of BMP-2 synthesized mineral-like nodules on the collagen fibers.

#### 3.4.4 Alizarin Red Staining

Mineralization was visualized by Alizarin red staining (Figure 7). First of all, no significant staining was detected in the C2C12, C2C12/BMP-2, and t-C2C12 groups. On the other hand, denser and larger mineralized areas were determined in the t-C2C12/BMP-2 group. It was concluded that transfection in the presence of BMP-2 induced mineralization compared to other groups.

BMP-2 is an osteogenic factor that induces cellular differentiation. Binding of BMP-2 to type I and type II receptors activates SMAD proteins. Following this, SMAD protein complex formed, and this complex regulates osteogenic genes.^52,53^ BMP-2, which is frequently used in bone tissue engineering studies, is the most potent osteo-inductive factor. It has a very short half-life and therefore requires high doses to differentiate cells. On the other hand, supra-physiological high dose of BMP-2 elicits inefficient bone composition or ectopic bone and cyst formation. At this point, internal stimulation of BMP-2 signaling in cells rather than using exogenous BMP-2 becomes an alternative approach.^54^*Noggin* controls BMP-2 activity as an antagonist. There is a feedback mechanism between BMP-2 and *Noggin*; BMP-2 synthesis induces *Noggin* production and *Noggin* blocks the BMP-2 function by binding to it.^55,56^ Neutralizing of *Noggin* with antibodies or small RNA molecules has been reported to promote osteogenesis.^17,18,56^ In a study, induction of BMP-2 expression and silencing of the *Noggin* gene with siRNA molecules synergistically increased osteogenic markers and mineralization.^57^ Fan et al. transfected ASCs (adipose-derived stem cells) with short hairpin RNA (shRNA) for knockdown of *Noggin* and seeded the transfected cells into BMP-2-loaded chitosan/chondroitin sulfate scaffolds. Transfection of *Noggin* shRNA significantly increased osteogenic differentiation of cells.^54^ Nguyen et al. encapsulated *siNoggin* molecules and MSCs into poly(ethylene glycol) hydrogels. Silencing *Noggin* stimulated *RUNX-2*, *BSP*, and *PPAR*-γ expressions. Besides ALP activity, total calcium amount and mineralization increased in cells.^58^ Cui et al. synthesized sterosomes to deliver *siNoggin* to cells. It was noted that the knockdown of *Noggin* increased *Runx-2, Alp*, and *Ocn* expression. Also, improved mineralization was observed via ALP and Alizarin red staining.^59^

Although this study suggests that the CRISPR/Cas9 system is a promising gene editing system due to its targeting of the coding and non-coding regions on the genome, this system has a number of limitations and risks. The main concerns regarding the implementation of CRISPR/Cas9-based gene editing are immunogenicity, non-targeting, polymorphism, delivery method, and ethics. Issues such as targeting immune-privileged organs, using bioinformatics tools, modification of Cas9 activity, designing multiple targets in a single cell, developing alternative delivery vectors such as liposomes, polymeric nanoparticles, and proposing ethical perspectives are currently being studied to overcome these limitations.^60,61^ In this context, in future studies, we aim to develop new delivery systems that will efficiently deliver CRISPR/Cas9 plasmids to various cells and enable high-throughput genome editing to support regeneration of different tissues.

## 4 Conclusions

To the best of our knowledge, this is the first *in vitro* study to investigate the cellular activities of CRISPR/Cas9-modified cells seeded on 3D scaffolds. Our findings demonstrated that: (i) a new cell source for bone tissue regeneration was obtained, since the DNA of CRISPR/Cas9-modified cells is permanently changed, (ii) BMP signaling was internally controlled by editing *Noggin* expression to reduce the required dose of BMP-2, (iii) *Noggin* knockout and BMP-2 synergistically induced osteogenic differentiation of cells, and (iv) the CRISPR/Cas9 system has been proposed as an alternative endogenous approach to the use of high-dose exogenous growth factor.

In conclusion, the combined use of *Noggin* suppression and controlled release of BMP-2 has great potential on osteogenic differentiation of cells for bone tissue regeneration.
